# Choi response criteria for early prediction of clinical outcome in patients with metastatic renal cell cancer treated with sunitinib

**DOI:** 10.1038/sj.bjc.6605567

**Published:** 2010-02-09

**Authors:** A A M van der Veldt, M R Meijerink, A J M van den Eertwegh, J B A G Haanen, E Boven

**Affiliations:** 1 Department of Medical Oncology, VU University Medical Center, Amsterdam, The Netherlands; 2 Department of Radiology, VU University Medical Center, Amsterdam, The Netherlands; 3 Department of Medical Oncology, The Netherlands Cancer Institute, Antoni van Leeuwenhoek Hospital, Amsterdam, The Netherlands

**Keywords:** sunitinib, renal cell cancer, Choi criteria, RECIST, tumour attenuation, tumour response

## Abstract

**Background:**

Because sunitinib can induce extensive necrosis in metastatic renal cell cancer (mRCC), we examined whether criteria defined by Choi might be valuable to predict early sunitinib efficacy.

**Methods:**

Computed tomography was used for measurement of tumour lesions in mm and lesion attenuation in Hounsfield units (HUs). According to Choi criteria partial response (PR) was defined as ⩾10% decrease in size or ⩾15% decrease in attenuation.

**Results:**

A total of 55 mRCC patients treated with sunitinib were included. At first evaluation, according to the Response Evaluation Criteria in Solid Tumours (RECIST) 7 patients had PR, 38 stable disease (SD), and 10 progressive disease (PD), whereas according to Choi criteria 36 patients had PR, 6 SD and 13 PD. Median tumour attenuation decreased from 66 to 47 HUs (*P*⩽0.001). In patients with PR, Choi criteria had a significantly better predictive value for progression-free survival and overall survival (both *P*s<0.001) than RECIST (*P*=0.685 and 0.191 respectively). The predictive value for RECIST increased (*P*=0.001 and <0.001 respectively), when best response during treatment was taken into account.

**Conclusion:**

Choi criteria could be helpful to define early mRCC patients who benefit from sunitinib, but the use of these criteria will not change the management of these patients.

The Response Evaluation Criteria in Solid Tumours (RECIST) is the most widely used measurement system in clinical trials and is based on the sum of the longest diameters of the appointed target lesions in the transversal plane (Therasse *et al*, 2000). Clinically meaningful responses, however, may be underestimated by RECIST, as new targeted therapies can cause tumour necrosis without a marked decrease in tumour size (Faivre *et al*, 2007). The receptor tyrosine kinase inhibitors (TKIs) imatinib, sunitinib, sorafenib, and axitinib are known to cause early and extensive necrosis (Vanel *et al*, 2005; Faivre *et al*, 2007; Flaherty, 2007; Rixe *et al*, 2007; Van der Veldt *et al*, 2008b). Treatment-induced necrosis, however, is not a part of RECIST and may even mimic progressive disease (PD).

In hepatocellular carcinoma (HCC), RECIST may be inappropriate as primary end point to evaluate sunitinib treatment (Faivre *et al*, 2007). Therefore, Faivre *et al* (2007) have used volumetric measurements for sunitinib-induced necrosis in HCC expressed as minor (<50%) and major (=50%) post-treatment tumour necrosis. As RECIST also underestimates imatinib-induced responses in gastrointestinal stromal cell tumours (GISTs), Choi *et al* (2007) have developed new response criteria to evaluate imatinib treatment in patients with GIST. These criteria include changes in tumour attenuation on computed tomography (CT), which reflect tumour density (Benjamin *et al*, 2007; Choi *et al*, 2007). Choi *et al* (2007) have defined a partial response (PR) as a ⩾10% decrease in one-dimensional tumour size or a ⩾15% decrease in tumour attenuation on CT scan, whereas PD was defined as ⩾10% increase in size without meeting PR criteria by change in attenuation (Table 1). The Choi criteria correlated better with disease-specific survival in imatinib-treated GIST patients than RECIST.

Several studies have indicated that sunitinib can induce necrosis in metastatic renal cell cancer (mRCC; Motzer *et al*, 2006; Baccala *et al*, 2007; Van der Veldt *et al*, 2008b). During sunitinib treatment, responding RCC lesions can be observed with dramatic decrease in attenuation, but little change in size. Although sunitinib has shown PR in 31% of patients in the first-line setting (Motzer *et al*, 2007), it remains unclear if RECIST optimally predicts treatment outcome and if new response criteria are required. Because the beneficial effect of anti-angiogenic agents in mRCC may also be stabilisation rather than substantial tumour regression in a large number of patients, the one-dimensional RECIST for PR, a ⩾30% decrease in the sum of the longest diameter of target lesions, may be inaccurate in the TKI era (Van Cruijsen *et al*, 2008). Because Choi criteria (Choi *et al*, 2007) may also be of value to evaluate tumour responses in tumours other than GIST, such as RCC and HCC, treated with targeted agents, we here compared the usefulness of Choi criteria with RECIST in sunitinib-treated mRCC patients.

## Patients and methods

### Patients and treatment

Medical records of patients were reviewed who were treated with sunitinib for advanced RCC in two centres in the Netherlands (VU University Medical Center and The Netherlands Cancer Institute) from December 2005 to October 2007. Most patients had been included in an expanded access programme (EAP) (Van der Veldt *et al*, 2008a) until September 2006 after which sunitinib was registered and available on doctor's prescription. In the EAP, each participant signed a protocol-specific informed consent approved by the institutional review board in accordance with national and institutional guidelines. For further analysis of CT scans according to Choi criteria, adequate safeguards to protect patient privacy were maintained.

Sunitinib was administered orally at a dose of 50 mg daily, consisting of 4 weeks of treatment followed by a 2-week rest period in cycles of 6 weeks. A dose reduction of sunitinib (to 37.5 or 25 mg) was allowed depending on the type and severity of adverse events. If patients had symptoms of PD during the rest period, there was the possibility for continuous dosing of sunitinib at 37.5 mg per day.

For evaluation of sunitinib efficacy, CT scans were performed at baseline and during treatment to assess clinical response according to RECIST version 1.0 (Therasse *et al*, 2000). For RECIST, best response was also determined on subsequent CT scans during treatment. Progression-free survival (PFS) was the time between the first day of sunitinib treatment and the date of PD on the CT scan according to RECIST, clear clinical evidence of PD, or death due to PD within 12 weeks after the last response evaluation. If a patient had not progressed, PFS was censored at the time of the last follow-up. If the PD date was unknown or a patient died due to PD later than 12 weeks after the last response evaluation, PFS was censored at the last adequate tumour assessment. Overall survival (OS) was the time between the first day of treatment and the date of death or the date at which a patient was last known to be alive. For PFS and OS analyses, data collection was closed on 1 September 2009.

### Image analysis

Patients were eligible for inclusion in the analysis, if they had CT scans at baseline and at first evaluation according to the same scan protocol in the same hospital and at least one tumour lesion at baseline >15 mm. Patients with bone metastasis (*n*=2) or primary tumour (*n*=1) as only evaluable lesion were excluded. Primary tumours were also excluded, as the overall response may be underestimated due to their enormous size (Van der Veldt *et al*, 2008b; Bex *et al*, 2009). Furthermore, brain (Helgason *et al*, 2008) and bone metastases at baseline were excluded.

Routine helical CT scans of the thorax and abdomen were obtained with a scanning delay of 30 and 70 s after start of intravenous (i.v.) injection of a low-osmolar non-ionic contrast agent (Omnipaque 300 (nycomed Amersham plc, Buckinghamshire, England) or Ultravist 300 (Bayer Shenng Pharma, Berlin, Germany)). For abdominal scans, Choi criteria were applied in the portal venous phase of contrast. All series were reconstructed in 5 mm contiguous axial slices. Scans were shown at standard soft tissue kernel and window (window centre, 20 Hounsfield units (HUs); window width, 360 HU) to avoid pixel averages from surrounding lung parenchyma. Image viewing and manipulation were controlled with Centricity RA 600 version 6.1 software (GE Healthcare Inc., Wauwatosa, WI, USA), which allows the radiologist to draw perimeters around the regions of interest. The software then automatically calculates the area enclosed by the perimeter and the mean attenuation of this area in HU. A specialised radiologist (MRM, 7 years of experience in radiology) masked to clinical history and patients' outcome and experienced in using the image viewing and manipulation software examined the CT scan images in the presence of a junior researcher (AAM vdV). To draw comparable perimeters over the tumours, we analysed CT scans at baseline and evaluation from one patient in the same session. Between the two observers, agreement on identification and delineation of the lesions was obtained in all cases. In addition, to evaluate the intra-observer variability for the determination of tumour attenuation, reproducibility of placing regions of interest over tumours was tested on 2 different days (>3 months between the measurements). As to the intra-observer variability for tumour attenuation measurements, Spearman's correlation coefficients were *ρ*⩾0.957 (*P*>0.001) for the HU value of individual lesions as well as the mean HU value in the individual patients.

For each patient, a maximum of 10 delineated tumour target lesions were identified (not more than 5 per organ). For RECIST measurements, the longest diameter of the tumour lesions was ⩾10 mm, whereas for Choi criteria the diameter was ⩾15 mm (Table 1; Choi *et al*, 2007). The attenuation on CT (density) of lesions ⩾15 mm was determined in HUs by drawing a region of interest around the margin of the entire tumour. Then, the tumour attenuation assessments of all lesions were combined and a mean attenuation on CT was computed for each patient. Thereafter, the percentage of change in attenuation from the pretreatment scan to the first evaluation during sunitinib was calculated for each patient.

### Statistics

Statistical analysis was performed using SPSS software (SPSS for Windows 15.0, SPSS Inc., Chicago, IL, USA). For testing possible correlations, the Spearman's correlation test was performed. The Wilcoxon's signed-ranks test was used to compare the changes in size and attenuation at baseline and at first evaluation. A two-tailed probability value of *P*<0.05 was considered significant. For the analyses according to RECIST and Choi criteria, patients were categorised into response (CR+PR) *vs* no response (SD+PD). For RECIST, patients were also classified as having clinical benefit (CR+PR and SD⩾12 weeks) *vs* no clinical benefit (SD<12 weeks and PD). PFS and OS were calculated with the Kaplan–Meier method. Log-rank test was used to test the differences between survival curves.

## Results

### Patients

A total of 55 mRCC patients treated with sunitinib were included in this study, of which 45 patients were participants in an EAP of sunitinib. One patient was excluded due to evident differences in phases of i.v. contrast between the two subsequent CT scans. Table 2 presents the patients' characteristics. The median age was 59 years (range: 20–81 years). Of 55, 48 patients had clear cell histology. Of 55 patients, 40 patients were cytokine-pretreated of whom 4 patients were also pretreated with other anti-angiogenic agents. The median time from the baseline CT scan and the initiation of sunitinib treatment was 0.5 months (range: 0–1.5 months). The median time from the start of sunitinib to the CT scan for first evaluation was 1.9 months (range: 1.1–3.4 months).

### Response according to RECIST

For RECIST measurements, 225 tumour lesions were eligible. At first evaluation these lesions showed a median change in tumour size of −10% (range: −100 to +189%). Seven (13%) patients reached PR, 38 (69%) patients stable disease (SD), and 10 (18%) patients PD, resulting in 7 responders and 48 non-responders. Five out of ten patients with PD were categorised as PD based on the occurrence of new lesions, including two patients with symptomatic brain metastases. Ten (18%) patients had SD at first evaluation, but reached a PR at later time points (median time to PR: 3.9 months; range: 2.4–9.7 months), resulting in an overall PR rate of 31%. A total of 24 patients had SD⩾12 weeks as best response.

### Response according to Choi criteria

**Figure 1:**
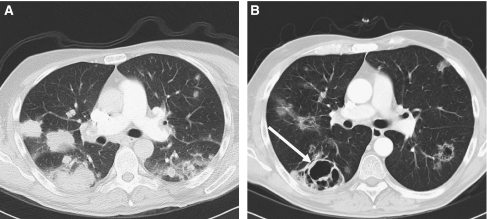
An example of a renal cell cancer patient with lung metastases at baseline (**A**) and pulmonary cavitations at first evaluation during sunitinib (**B**, arrow). For the purpose of illustration the lung window setting is shown.

**Figure 2:**
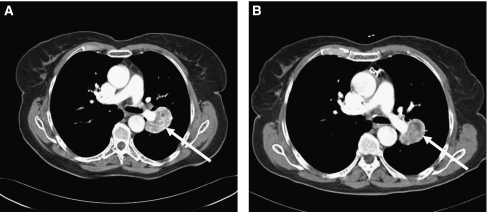
An example of a renal cell cancer patient on sunitinib treatment in which the lung lesion (arrow) showed a decrease in attenuation at first evaluation. (**A**) At baseline, the tumour attenuation was 107 Hounsfield units (HUs) and the longest diameter 48 mm. (**B**) At first evaluation, the tumour attenuation was 65 HUs (−39%) and the longest diameter was 39 mm (−19%).

For Choi criteria, less tumour lesions were eligible than for RECIST (Table 3; Figure 1), namely 173. Lesions most frequently excluded from the analysis had a tumour size <15 mm before treatment (*n*=38). In 24 lesions the change in tumour attenuation could not be determined due to a tumour size <15 mm at evaluation. At baseline, the median tumour size was 26 mm (range: 15–140 mm) for all lesions, with a median attenuation of 66 HUs (range: 6–135 HUs). During sunitinib at first evaluation, the median size and attenuation had decreased to, respectively, 24 mm (range: 0–186 mm; Wilcoxon's signed-ranks test, *P*⩽0.001) and 47 HUs (range: 4–112 HUs; *P*⩽0.001). A significant decrease in attenuation was measured at all tumour sites (Table 4; Figure 2). Preliminary analysis did not show a significant difference in the change in attenuation between the seven patients with non-clear cell histology and the 48 patients with clear cell histology. Overall, a weak correlation was calculated between the percentage of change in tumour size and the percentage of change in attenuation (Spearman's *ρ*=0.187, *P*=0.022).

At first evaluation according to Choi criteria, 36 (65%) patients reached PR, 6 (11%) had SD, and 13 (24%) had PD, resulting in 36 responders and 19 non-responders. Of 38, 29 patients who were categorised as SD according to RECIST had PR according to Choi criteria. Patients were categorised as PR according to Choi based on decrease in tumour size ⩾10% (*n*=12), decrease in tumour attenuation ⩾15% (*n*=9) or both (*n*=15). All six patients with SD according to Choi criteria had a PFS⩾12 weeks. Of note, 3 out of 38 patients defined as SD by RECIST had PD according to Choi criteria. These three patients had a PFS>12 weeks among whom was one patient that reached a PFS>10 months.

### Survival analysis

**Figure 3:**
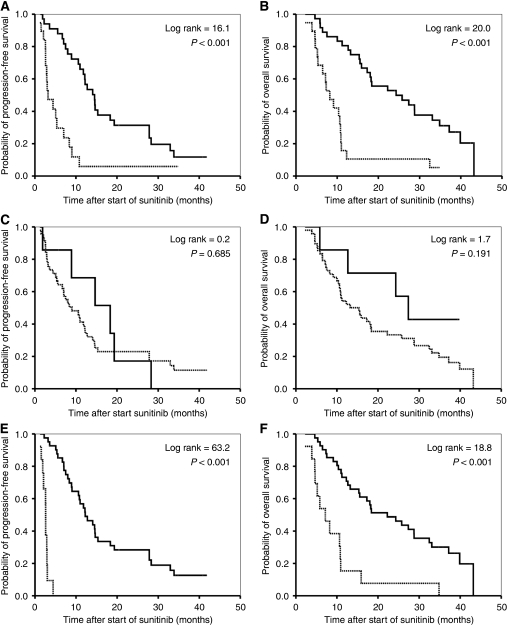
Kaplan–Meier curves for progression-free survival and overall survival of patients with metastatic renal cell cancer treated with sunitinib: responders (—) and non-responders (- - -) at first evaluation according to Choi criteria (**A**, **B**) and the Response Evaluation Criteria in Solid Tumours (RECIST) (**C**, **D**) as well as clinical benefit (partial response+stable disease ⩾12 weeks) (—) and no clinical benefit (progressive disease+stable disease <12 weeks) (- - -) at first evaluation by RECIST (**E**, **F**).

At first evaluation in patients with PR, Choi criteria had a significantly better predictive value for PFS and OS (*P*<0.001 for both) than RECIST (*P*=0.685 and 0.191 respectively) (Table 5; Figure 3). When best response during treatment was analysed according to RECIST, the predictive value of RECIST increased for both PFS and OS (*P*=0.001 and <0.001 respectively). For clinical benefit (PR and SD⩾12 weeks), the predictive value of RECIST for PFS and OS was also significant (*P*<0.001 for both). When the two Choi criteria were analysed separately, in which patients with new lesions were categorised as PD, only a 15% decrease in attenuation was predictive for PFS (*P*=0.018; log rank=5.6) and OS (*P*=0.005; log rank=7.8).

## Discussion

In this study, we evaluated whether the new Choi criteria, which include changes in tumour attenuation, are of additional value to predict outcome in mRCC patients treated with sunitinib. The response rate of 31% as measured by RECIST was comparable with that reported previously (Motzer *et al*, 2007), indicating that the present study is representative for sunitinib treatment in mRCC patients. At first evaluation, Choi criteria of PR were able to define a large population with a long PFS and OS, whereas RECIST PR only identified seven patients with favourable clinical outcome. When patients with PR and SD⩾12 weeks during treatment were taken into account, the predictive value of RECIST substantially increased. The latter could have been expected, as patients with SD at first evaluation are likely to continue sunitinib treatment and a substantial number will eventually reach a PR or SD⩾12 weeks.

During sunitinib treatment, we observed tumour necrosis as illustrated by a reduction in tumour attenuation with a median decrease of 24% at first evaluation. The TKI sorafenib can also induce extensive necrosis (Flaherty, 2007). In comparison with placebo, sorafenib prolonged PFS in cytokine-pretreated mRCC with almost 3 months (Escudier *et al*, 2007), although the complete response (CR) and PR rates by RECIST were only <1 and 10% respectively (Escudier *et al*, 2007). These data suggest that non-responders by RECIST may have benefited from sorafenib. Therefore, Choi criteria may also be valuable to early identify mRCC patients who will have a favourable outcome from sorafenib treatment. Response evaluation by Choi criteria should ideally be planned on a fixed day during treatment, i.e. day 28 of sunitinib administration in the 4 weeks on 2 weeks off schedule in mRCC. The variability in timing of the first on-treatment CT scan in this study, however, could not be avoided because of its retrospective design.

Although Choi criteria could easily be applied on routine standardised contrast-enhanced CT scans, there were several limitations in the use of these criteria for evaluation of sunitinib-induced responses in mRCC. First, a large number of lesions (22%), especially lung metastases, which could be measured by RECIST, had to be excluded due to a size <15 mm at baseline. As mRCC patients can have rather small metastases, the reliability of Choi criteria may decrease when smaller lesions (<15 mm) significantly decrease in size or attenuation, but are ineligible for assessment. Second, the reliability of Choi criteria may also decrease when fewer lesions are included for analysis. For example, a 15% decrease in attenuation in five lesions would be more reasonable than measured in one single lesion. Third, the attenuation of heterogeneous lesions may be assessed inaccurately, as the mean value is calculated in one region of interest in one slice. Fourth, measurements of relatively hypodense lesions at baseline may be less reliable, because a 15% decrease in attenuation is less accurate than measurements in lesions with higher pretreatment attenuation values. For that reason, use of absolute change may be more precise than the percent change in attenuation. Fifth, attenuation measurements are not possible in lesions with sunitinib-induced cavitations, which was the case in eight lung lesions (Figure 1). Sixth, although the intra-observer variability appeared to be rather low, the above-described limitations of Choi criteria imply a risk of high inter-observer variability and may even lead to a change in response in an individual patient. Therefore, mutual agreement on the delineation method should be achieved between the observers. Last, although i.v. contrast was administered according to the same scanning protocol and patients with clear differences in i.v. contrast were excluded, slightly different phases of scanning at subsequent time points may lead to incorrect changes in lesion attenuation. In that respect, it should also be mentioned that sunitinib-induced changes in cardiac output (Chu *et al*, 2007) might possibly influence the distribution of i.v. contrast. In mRCC patients, administration of i.v. contrast may be harmful in the presence of an impaired renal function.

The ultimate goal in the palliative treatment of mRCC with sunitinib is prevention of disease progression combined with acceptable quality of life. Sunitinib, however, is associated with a wide range of mild toxicities that can be cumbersome, and alternative treatments for mRCC, such as sorafenib and temsirolimus, are readily available. Therefore, surrogate markers for poor PFS and OS are warranted in patients with SD at first evaluation. Unfortunately, Choi criteria were not able to identify patients with clear-cut progression, because three patients defined as PD had a PFS>12 weeks. Compared with RECIST, Choi criteria may be less optimal for identifying PD, probably due to the ⩾10% increase *vs* the ⩾20% increase used by RECIST.

In conclusion, Choi criteria can be easily applied on contrast-enhanced CT scans of mRCC patients treated with sunitinib, but its reliability is limited, especially in patients with most lesions <15 mm, a small number of lesions, heterogeneous lesions or hypodense lesions at baseline. Although Choi criteria had a significantly better predictive value for PFS and OS than RECIST at first evaluation in patients with PR, its predictive value for outcome was similar to that of RECIST at later time points. Because Choi criteria were not able to early identify patients with PD, these criteria will not change the management of sunitinib-treated mRCC patients.

## Figures and Tables

**Table 1:** Choi response criteria (Choi *et al*, 2007)

**Response**	**Definition**
CR	Disappearance of all lesions
	No new lesions
PR	A decrease in size ⩾10% or a decrease in tumour attenuation (HU) ⩾15% on CT
	No new lesions
	No obvious progression of non-measurable disease
SD	Does not meet criteria for CR, PR, or PD
	No symptomatic deterioration attributed to tumour progression
PD	An increase in tumour size ⩾10% and does not meet criteria of PR by tumour attenuation on CT
	New lesions

Abbreviations: CR=complete response; PR=partial response; SD=stable disease; PD=progressive disease; HU=Hounsfield unit.

**Table 2:** Patient characteristics

	**Total *N*=55**
**Variable**	***N* (%)**
*Sex*	
Male	34 (62)
Female	21 (38)
	
Median age, years (range)	59 (20–81)
	
*ECOG performance status*	
0	24 (43)
1	22 (40)
2	5 (9)
3	2 (4)
Unknown	2 (4)
	
*Tumour type*	
Clear cell	48 (87)
Other	7 (13)
	
Previous nephrectomy	45 (82)
	
*Prior treatment*	
None	15 (27)
Cytokine based-therapy	40 (73)
Anti-angiogenic therapy	4 (7)
	
*No. of disease sites*	
1	7(13)
2	20 (36)
⩾3	28 (51)
	
*Sites of disease*	
Lung	49 (89)
Lymph nodes	32 (58)
Bone	13 (24)
Liver	11 (20)
Local recurrence	7 (13)
Brain	4 (7)
	
*MSKCC risk groups* a	
0 (favourable)	11 (20)
1–2 (intermediate)	36 (65)
⩾3 (poor)	7 (13)
Unknown	1 (2)

Abbreviations: ECOG=Eastern Cooperative Oncology Group; MSKCC=Memorial Sloan-Kettering Cancer Center.

a Risk groups according to MSKCC prognostic criteria (based on the 5 risk factors: low Karnofsky performance status (<80%), high LDH (>1.5 times the upper limit of normal), low serum haemoglobin, high corrected serum calcium (>10 mg per 100 ml), and time from initial diagnosis to treatment of less than 1 year) (Motzer *et al*, 2002).

**Table 3:** Tumour lesions for the efficacy analysis in patients with mRCC treated with sunitinib

	***N***
*Eligible lesions for analysis*	
RECIST	225
Choi criteria	173
	
*Exclusion according to both RECIST and Choi criteria*	26
Bone metastasis	11
Primary tumour	10
Brain metastasis	5
	
*Exclusion according to Choi criteria only*	52
10 mm ⩽ tumour lesion at baseline <15 mm	38
Air-containing cavity at evaluation	8
Beam-hardening artefact obscuring helical CT density (e.g. metal-containing parts)	6

Abbreviations: RECIST=Response Evaluation Criteria in Solid Tumours; CT=computed tomography.

**Table 4:** Change in tumour size and density for tumour lesions included in the Choi criteria for evaluation of sunitinib treatment in patients with mRCC

	**Number of eligible lesions^a^**	**Median pretreatment values (range)**		**Median values at first evaluation (range)**		**Median change (range)**	
**Tumour site**	***N* (size/attenuation)**	**Size (mm)**	**Attenuation (HUs)**	**Size (mm)**	**Attenuation (HUs)**	**Size (%)**	**Attenuation (%)**
Lung	55/41	25 (15–91)	59 (15–98)	19 (5–110)^**^	38 (4–81)^**^	−24 (−71 to +21)	−31 (−92 to +146)
Lymph node	63/56	25 (15–123)	68 (6–118)	25 (9–101)	55 (17–105)^**^	−5 (−57 to +59)	−14 (−69 to +183)
Liver	16/16	31 (19–83)	83 (40–96)	32 (16–67)	51 (22–66)^**^	−3 (−25 to +45)	−38 (−76 to −5)
Abdominal other^b^	26/24	41 (15–140)	65 (33–135)	41 (0–186)	61 (20–112)^*^	−4 (−100 to +65)	−13 (−78 to +47)
Thoracic other^c^	13/12	27 (17–44)	67 (18–116)	20 (13–55)	53 (21–87)	−23 (−39 to +189)	−19 (−73 to +28)
Total number of lesions	173/149	26 (15–140)	66 (6–135)	24 (0–186)^**^	47 (4–112)^**^	−11 (−100 to +189)	−24 (−92 to +183)

Abbreviation: HU=Hounsfield unit.

^*^P⩽0.05, ^**^P⩽0.001 compared to baseline value by the Wilcoxon's signed-ranks test.

a Tumour lesions ⩾15 mm at baseline but <15 mm at evaluation were included for the analyses regarding the change in tumour size, but were excluded for the analyses regarding the change in tumour attenuation.

b Abdominal sites, including local recurrence, adrenal gland, spleen, and peritoneum.

c Thoracic sites including pleura, breast, and subcutis.

**Table 5:** PFS and OS of mRCC according to RECIST and Choi criteria

	**Median PFS^a^**	**Median OS**
	**Months (range)**	**Months (range)**
*Response*		
Choi criteria	Log rank=16.1, *P*<0.001	Log rank=20.0, *P*<0.001
Responders^b^ (*n*=36)	14.5	25.4
Non-responders^c^ (*n*=19)	3.2	10.4
		
RECIST at first evaluation	Log rank=0.2, *P*=0.685	Log rank=1.7, *P*=0.191
Responders^b^ (*n*=7)	18.3	27.4
Non-responders^c^ (*n*=48)	9.0	13.2
		
RECIST at best response	Log rank=11.2, *P*=0.001	Log rank=13.2, *P*<0.001
Responders^b^ (*n*=17)	19.3	31.3
Non-responders^c^ (*n*=38)	7.0	15.3
		
*Clinical benefit*		
RECIST	Log rank=63.2, *P*<0.001	Log rank=18.8, *P*<0.001
Clinical benefit^d^ (*n*=41)^a^	12.2	22.3
No clinical benefit^e^ (*n*=13)	2.6	7.2

Abbreviations: RECIST=Response Evaluation Criteria in Solid Tumours; PFS=progression-free survival; OS=overall survival.

a For one patient with stable disease, date of progressive disease was not available.

b Partial response.

c Stable disease+progressive disease.

d Partial response+stable disease ⩾12 weeks.

e Progressive disease+stable disease <12 weeks.
